# Supplementary material for: Further Studies on the 3-Ketosteroid 9α-Hydroxylase of Rhodococcus ruber Chol-4, a Rieske Oxygenase of the Steroid Degradation Pathway
Source: Microorganisms. 2021 May 29;9(6):1171. doi: 10.3390/microorganisms9061171 (PMC8228715; doi:10.3390/microorganisms9061171)
Supplement: Supplementary file 1 [file microorganisms-09-01171-s001.zip › microorganisms-1219156-supplementary.pdf]

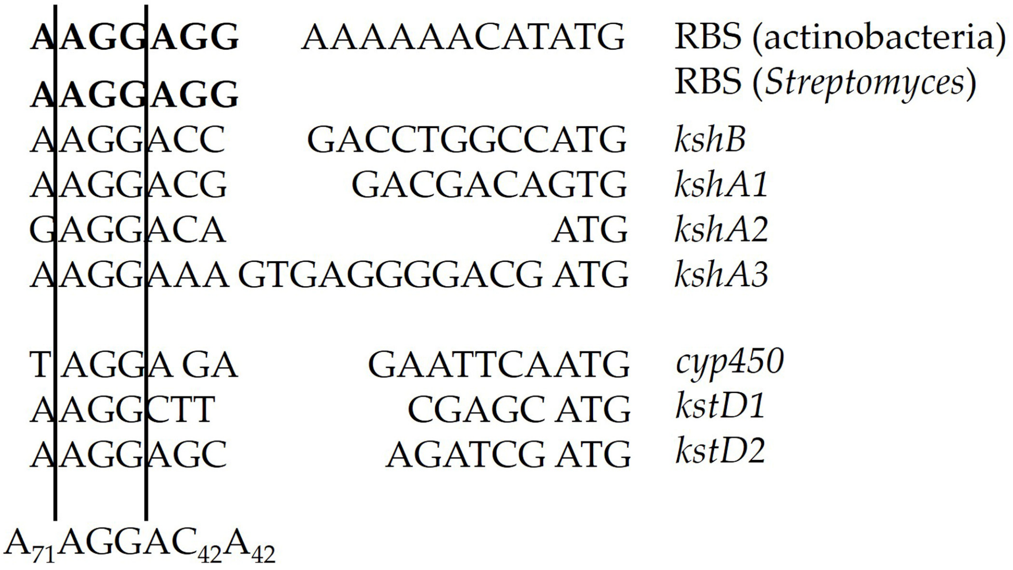


**Figure S1.** RBS comparison of some steroid *Rhodococcus ruber* genes. The Figure 41. or actinobacteria [42]. The rest lines are ORFs from *R. ruber* related with the steroid catabolism. The nucleotides before the initial codon are also indicated. The proposed consensus takes into account only the *R. ruber* putative RBS.

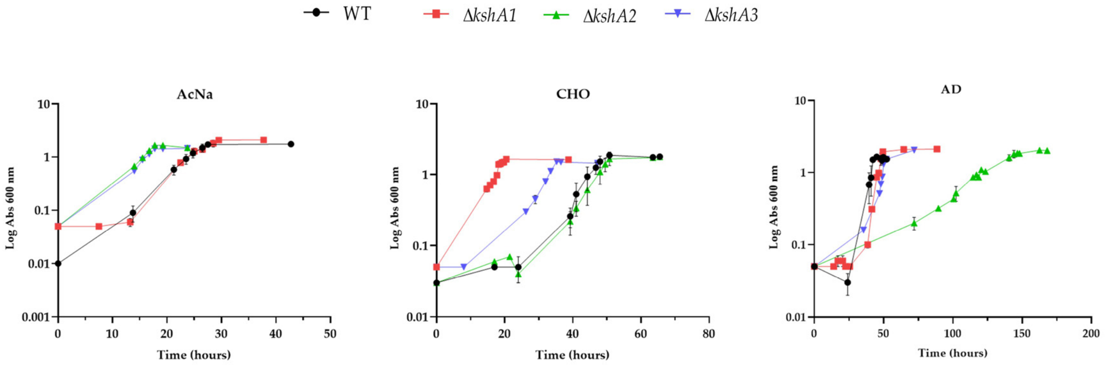


**Figure S2.** Growth curves of WT and *ksh* single mutants *Rhodococcus ruber* strains in minimal medium with different steroids as carbon source. A representative experiment of 3–6 replicates is shown.

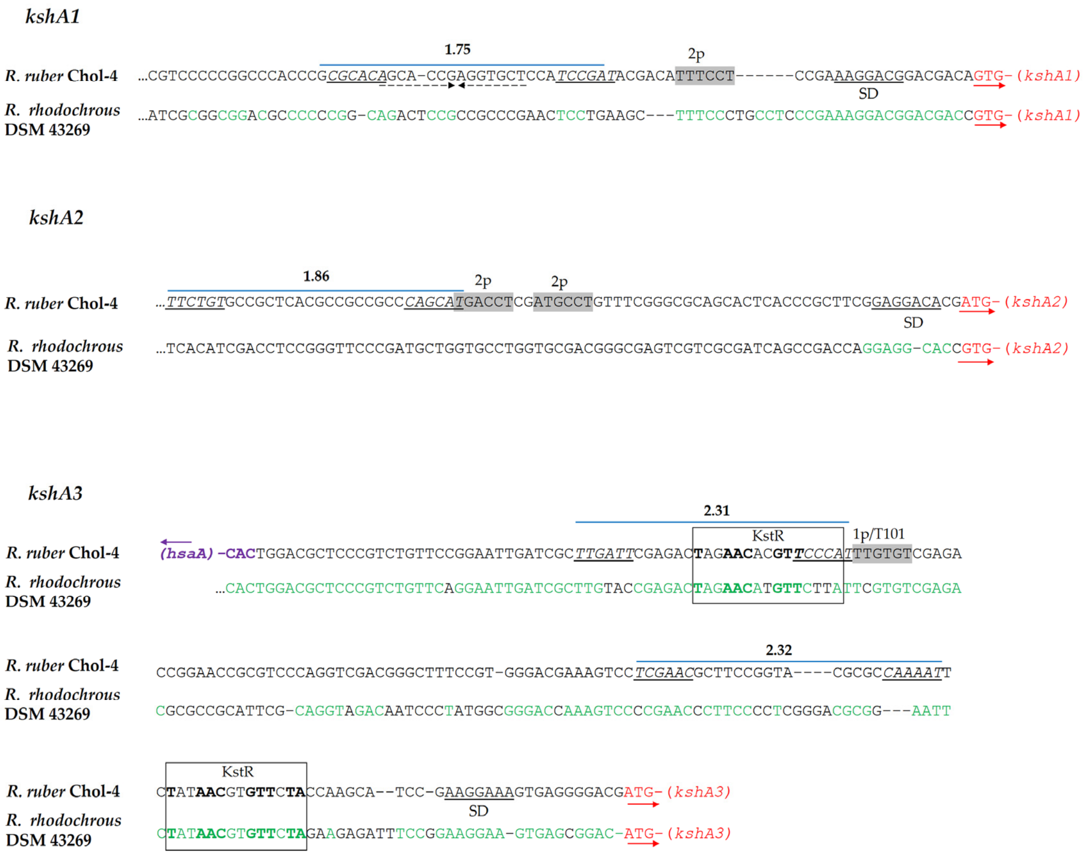


**Figure S3.** Comparison of upstream regions of *ksh* ORFs. Data accession: *R. ruber* (NCIB::ANGC00000000.2), HQ425873 (*kshA1*), HQ425874 (*kshA2*), HQ425875 (*kshA3*), HQ425876 (*kshA4*), and HQ425877 (*kshA5*).
